# Global scientific trends on neuroimaging in obsessive-compulsive disorder in the early twenty-first century: a bibliometric analysis and visualization analysis

**DOI:** 10.1186/s12991-026-00653-6

**Published:** 2026-03-31

**Authors:** Cong Zhou, Yan Fan, Aoxue Zhang, Xiaodong Cheng, Jian Cui, Kun Li, Chuanxin Liu, Hao Yu, Sen Li

**Affiliations:** 1 School of Mental Health, Jining Medical University, Jining, China; 2 Department of Psychology, Affiliated Hospital of Jining Medical University, Jining, China; 3 Department of Psychiatry, Shandong Daizhuang Hospital, Jining, China

**Keywords:** Obsessive-compulsive disorder, Neuroimaging, Bibliometric analysis, Functional magnetic resonance imaging, Transdiagnostic biomarkers

## Abstract

**Objective:**

This bibliometric analysis aims to delineate the evolution, current trends, and future directions in neuroimaging research on obsessive-compulsive disorder (OCD) from 2000 to 2024. By synthesizing global research output, collaboration networks, and technological advancements, we seek to highlight knowledge gaps and propose strategies to enhance the equity, reproducibility, and translational impact of OCD neuroimaging studies.

**Methods:**

Data were sourced from the Web of Science Core Collection, spanning January 1, 2000, to December 31, 2024. A structured search strategy identified 2,000 eligible articles and reviews. Bibliometric analysis was conducted using CiteSpace, VOSviewer, and Excel to examine publication trends, geographic and institutional collaborations, keyword co-occurrence, citation patterns, and journal influence.

**Results:**

OCD neuroimaging research has shown sustained growth, with a shift from metabolic studies to network neuroscience, driven by advances in functional magnetic resonance imaging (fMRI), diffusion tensor imaging (DTI), and artificial intelligence (AI). High-income countries dominated research output, with the United States contributing 36.5% of publications. However, low- and middle-income countries (LMICs) remain underrepresented. Keyword analysis revealed emerging themes such as transdiagnostic biomarkers and neuromodulation therapies. High-impact journals like *Biological Psychiatry* and *American Journal of Psychiatry* dominated citations, while specialized journals struggled with visibility. Methodological heterogeneity and limited data sharing posed significant challenges to reproducibility.

**Conclusion:**

This bibliometric analysis maps the transformation of OCD neuroimaging into a central pillar of translational psychiatry, revealing sustained growth, a paradigm shift toward network-based methods, and persistent geographical disparities. These documented trends collectively highlight that advancing equitable and reproducible research requires prioritized global collaboration and the adoption of open science frameworks. Therefore, addressing these specific gaps is crucial for developing neuroimaging-informed biomarkers and interventions with broad clinical applicability.

**Supplementary Information:**

The online version contains supplementary material available at 10.1186/s12991-026-00653-6.

## Introduction

Obsessive-compulsive disorder (OCD) is a severe psychiatric disorder characterized by persistent intrusive thoughts and repetitive ritualistic behaviors that significantly impair daily functioning [1]. Beyond individual suffering, these symptoms disrupt social relationships, academic and occupational performance, and family dynamics. Epidemiological studies estimate the lifetime prevalence of OCD at 2–3% in the general population [2]. Although research has advanced, the disorder’s exact etiology remains unclear, with evidence suggesting a complex interplay of neurobiological, genetic, and environmental factors. Dysregulation of neurotransmitters—particularly serotonin, dopamine, and glutamate—has been consistently linked to OCD pathogenesis [1], while external stressors, life events, and cognitive vulnerabilities may exacerbate symptom manifestation and progression [3].

In recent years, breakthroughs in neuroimaging technologies, such as functional magnetic resonance imaging (fMRI), diffusion tensor imaging (DTI), and positron emission tomography (PET)— have revolutionized OCD research by elucidating its neurobiological underpinnings. These studies highlighted disruptions in cortico-striato-thalamo-cortical (CSTC) circuits, a key pathway in OCD pathophysiology [4]. For instance, pioneering work by Menzies et al. [5] integrated neuroimaging and neuropsychological data to refine the orbitofronto-striatal model of OCD, revealing disrupted connectivity in frontostriatal pathways. Milad et al. [6] further extended the model by implicating broader cortical networks in cognitive inflexibility and decision-making deficits. Complementing functional and metabolic approaches, structural magnetic resonance imaging (sMRI) has been pivotal in delineating the neuroanatomical correlates of obsessive-compulsive disorder. Voxel-based morphometry (VBM) studies have identified a pattern of increased grey matter volume in the thalamus and striatum alongside decreased volume in the medial prefrontal/anterior cingulate cortex and inferior frontal gyrus [7, 8]. Investigations into cortical thickness reveal a complex profile, with thinning observed in the anterior cingulate and insula regions, and thickening in areas such as the lingual gyrus and regions of the frontal lobe [9]. The inconsistency of early, small-scale studies has been effectively addressed by large-scale, worldwide collaborative efforts. Meta- and mega-analyses led by the ENIGMA OCD working group have also provided high-powered evidence for distinct neurodevelopmental trajectories [10–15]. Critically, a multimodal meta-analysis integrating structural and functional data confirms that core regions like the medial prefrontal/anterior cingulate cortex and insula exhibit convergent abnormalities in both grey matter structure and intrinsic function, highlighting them as central nodes in the pathophysiology of obsessive-compulsive disorder [7]. These robust structural findings extend beyond the classic CSTC circuit model, underscoring the anatomical complexity of the disorder and providing a solid foundation for the integrative, multimodal research landscape analyzed in the present bibliometric study.

Despite these advances, the field lacks a systematic synthesis of global research trends, including collaborative networks, technological innovations, and knowledge dissemination in the 21 st century. Existing reviews often focus narrowly on specific neuroimaging modalities or regional findings, overlooking the broader evolution of interdisciplinary collaboration and global research dissemination. A comprehensive bibliometric analysis is thus urgently needed to map the trajectory of OCD neuroimaging research, identify key contributors, and highlight emerging priorities. The exponential growth of OCD neuroimaging research—with over 2,000 publications indexed in the Web of Science Core Collection from 2000 to 2024 —presents both challenges and opportunities. While narrative reviews have effectively explored specific neurobiological mechanisms, they fail to objectively quantify broader trends, such as thematic shifts, geographic distribution, and institutional contributions. For example, early studies in the 1980 s on glucose metabolism revealed critical metabolic abnormalities in OCD [16], yet newer methodologies like connectomics and machine learning-enhanced neuroimaging remain unclarified in bibliometric studies [17, 18]. The publication landscape for OCD research is also highly fragmented. Although specialized journals like the *Journal of Obsessive-Compulsive and Related Disorders* focus on OCD spectrum disorders, their impact is limited (impact factor: 1.9, Q3 in JCR 2024), with high-impact research often published in broader psychiatry or neuroscience journals. This dispersion underscores the need for a bibliometric analysis to evaluate the field’s knowledge structure, collaborative patterns, and emerging priorities thereby mapping the evolution of neuroimaging research and identifying key technological advancements in the 21 st century.

Geopolitical and socioeconomic disparities further complicate OCD neuroimaging research. Most high-impact studies originate from high-income countries, with collaboration networks dominated by Western institutions [14, 19]. This bias risks exacerbating inequities in knowledge production and clinical translation. For instance, while the United States and Europe lead in publication output, contributions from low- and middle-income countries (LMICs) —where OCD prevalence is similar but resources are scarce—are frequently marginalized. Additionally, while resting-state fMRI and artificial intelligence (AI)-driven tools have transformed research paradigms since 2010 [18], their adoption rates across regions remain unstudied. Without a comprehensive analysis of these trends, the field may overlook underrepresented methodologies and populations, hindering the development of globally relevant diagnostic and therapeutic strategies. This highlights the need for a systematic evaluation of the field’s knowledge structure and collaborative dynamics to ensure equitable progress in OCD neuroimaging research.

To address these gaps, this study employs bibliometric methods to analyze the OCD neuroimaging literature from 2000 to 2024. Using data from the Web of Science Core Collection, we identify temporal and thematic trends-from early metabolic studies to contemporary network neuroscience-and assess the impact of technological advancements. We also map global collaboration networks, analyze keyword co-occurrence and citation bursts, and evaluate journal influence and interdisciplinary crossover. These findings will help forecast emerging frontiers and inform equitable, globally applicable diagnostic and therapeutic approaches. Translating this goal into practice requires a concerted effort across multiple fronts. A foundational step is the commitment to large-scale, open-science collaborations, such as the ENIGMA-OCD project, which harmonizes data across diverse global populations to discover reproducible biosignatures and ensure generalizable findings [11]. Concurrently, improving global access to evidence-based care necessitates scaling up therapist training through structured programs like the Behavior Therapy Training Institute (BTTI) and leveraging digital health platforms for delivery, guided by established clinical guidelines [20]. Ultimately, advancing towards personalized medicine depends on prioritizing the development of clinically translatable predictive tools, where integrating multimodal data with advanced computational methods like deep learning holds promise for forecasting individual treatment outcomes and guiding precise intervention [21].

## Materials and methods

### Data source and search strategy

**Fig. 1:**
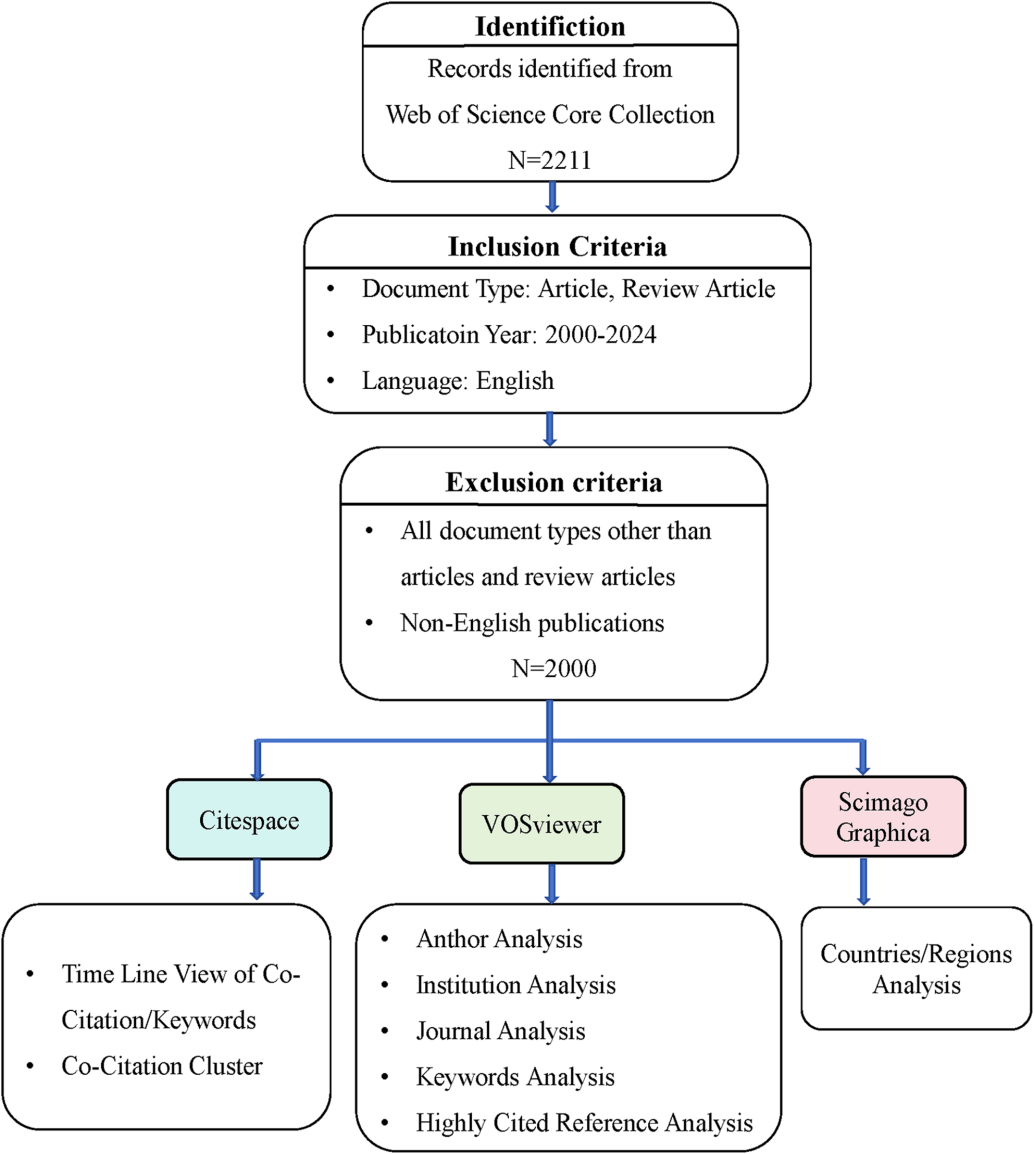
PRISMA flow diagram of study identification, screening, and inclusion for bibliometric analysis

All literature data in this study were sourced from the Web of Science Core Collection database, which encompasses academic publications across nearly 300 disciplines worldwide and is widely utilized by researchers for bibliometric analyses in various fields [22, 23]. The bibliometric analysis conducted in this study spans the period from January 1, 2000, to December 31, 2024. The search strategy employed was as follows: in the Web of Science Advanced Search, the query string (TI=(“obsessive-compulsive disorder*” OR “obsessive compulsive disorder*” OR “OCD”) OR AK=(“obsessive-compulsive disorder*” OR “obsessive compulsive disorder*” OR “OCD”) OR AB=(“obsessive-compulsive disorder*” OR “obsessive compulsive disorder*” OR “OCD”)) AND (TS=(“neuroimaging” OR “MRI” OR “magnetic resonance imaging” OR “fMRI” OR “functional magnetic resonance imaging” OR “sMRI” OR “structural magnetic resonance imaging” OR “DTI” OR “diffusion tensor imaging” OR “3D-T1” OR “EEG” OR “electroencephalogram” OR “ERP” OR “event related potential” OR “PET imaging” OR “fluorodeoxyglucose positron emission tomography-computed tomography (18FDG-PET-CT)” OR “Positron emission tomography-computed tomography scan”)) was entered. This search yielded 2,211 articles, from which publications dated beyond 2025 were excluded. Only articles and review articles published between 2000 and 2024 and written in English were selected, resulting in a final dataset of 2,000 eligible publications. As shown in Fig. 1.

### Data analysis

After verifying the accuracy and validity of the data, we exported the refined and optimized raw dataset in plain text format, which included critical information such as titles, authors, keywords, institutions, countries/regions, citations, journals, and publication dates. Subsequently, we utilized Microsoft Office Excel 2021, VOSviewer (version 1.6.18), and CiteSpace (version 6.1.R6) as primary tools for data analysis and visualization.

CiteSpace [24, 25], developed by Chaomei Chen and colleagues, is a bibliometric software designed to generate domain-specific network maps and extract key insights from research, such as emerging trends, hotspots, and directions. In this study, we employed this software to analyze co-occurrence and clustering patterns among authors, research institutions, and countries. Additionally, we used VOSviewer [26, 27], a Java-based software developed by Nees Jan van Eck and colleagues in 2010, which is widely applicable for visualizing and analyzing bibliometric data. VOSviewer was utilized to examine the distribution of countries/regions, institutions, collaborative networks among authors, as well as the distribution and relationships of keywords. To ensure the robustness and interpretability of the keyword co-occurrence analysis, a standardized data preprocessing protocol was implemented. First, the analysis was exclusively based on Author Keywords provided in the original publications to preserve author intent and ensure semantic precision. Second, all extracted keywords underwent a standardization process to merge synonyms and reduce redundancy. This included unifying singular and plural forms, harmonizing spelling variants (e.g., “fMRI” and “functional magnetic resonance imaging”), and merging conceptually equivalent terms. Third, to construct a meaningful and interpretable co-occurrence network, a minimum occurrence threshold was applied. Keywords were included in the final network only if they appeared at least 15 times across the corpus. This threshold was determined empirically through preliminary analyses to balance the inclusiveness of meaningful themes and the clarity of the network structure, aligning with common practices in bibliometric research.

## Results

### Publication and citation analysis

**Fig. 2:**
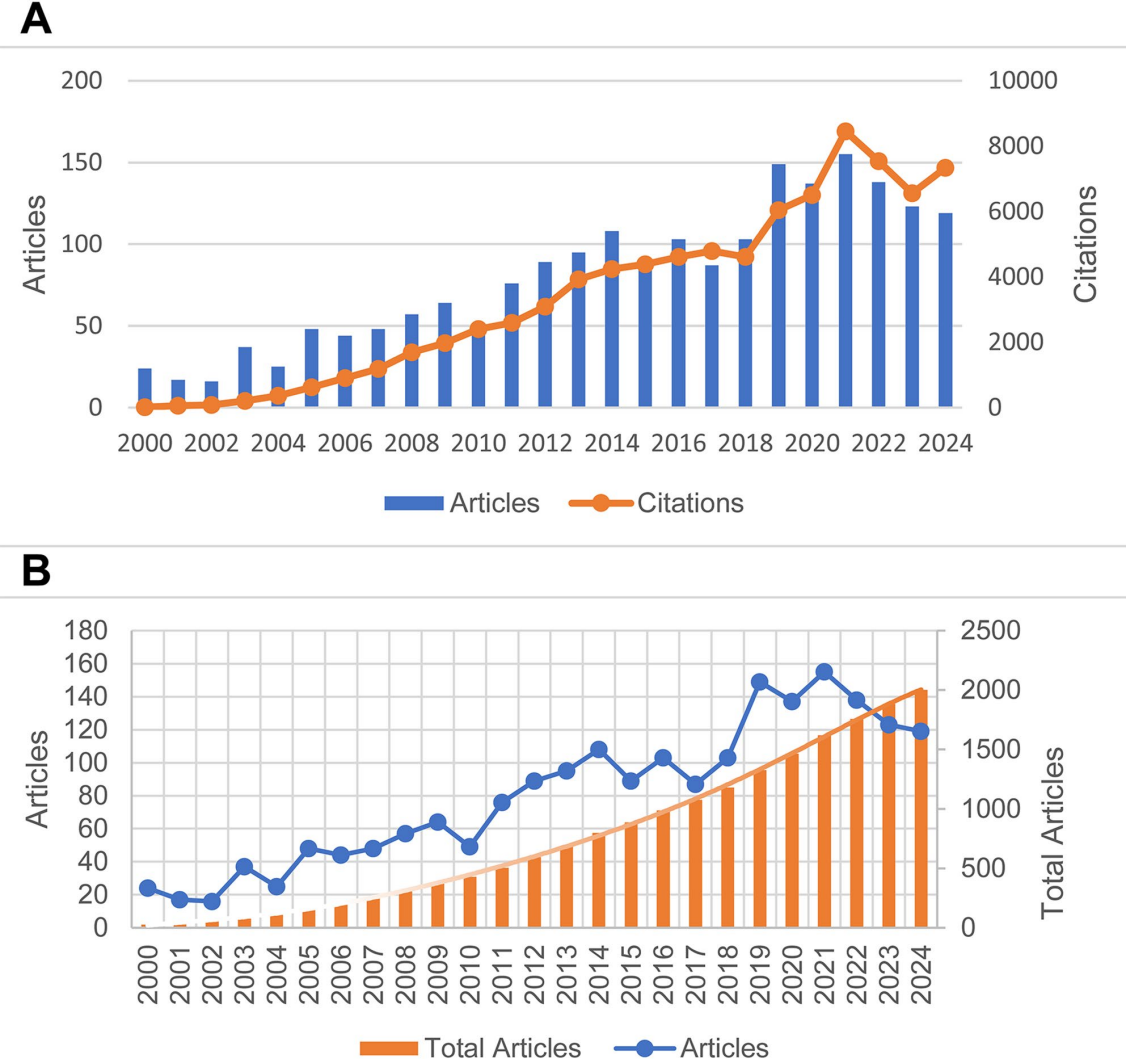
Temporal dynamics of scholarly output and citation impact in the field of OCD from 2000 to 2024. (**A**) Annual publication volume and citation frequency within the field of OCD spanning the period from 2000 to 2024. (**B**) Annual publication volume, cumulative publication totals, and their corresponding polynomial trend lines for Patent Foramen Ovale research conducted between 2000 and 2024

Figure 2 illustrates the trends in the number of publications and citations in the field of OCD from 2000 to 2024. In Fig. 2A, the blue bars represent the annual number of publications, while the orange line indicates the number of citations. The data reveal that both the publication output and citation frequency exhibited a fluctuating upward trend over the 14-year period prior to 2021, reaching a peak in that year (155 publications, 8,457 citations). This may suggest that 2021 was a year of significant academic breakthroughs and concentrated research output in the field of OCD. Although the number of publications declined between 2021 and 2024, the overall quantity remained substantial. Notably, the citation frequency began to rise again in 2024. The progressively increasing orange bars in Fig. 2B demonstrate that new articles have been continuously published in this field, with the total number of articles rising annually. The years with higher publication outputs show more pronounced increases.

The ratio of citations to publications showed a steady increase over time, indicating a progressive enhancement in the representativeness and academic impact of neuroimaging research on obsessive-compulsive disorder. Notably, several periods with moderate publication output, particularly between 2006 and 2009, exhibited disproportionately high citation-to-publication ratios, highlighting the presence of highly influential and potentially foundational studies. Furthermore, multiple years demonstrated exceptionally high citation efficiency, with the citation-to-publication ratio exceeding 50 in selected years (e.g., 2015, 2017, and 2021–2024), indicating sustained high average impact per article. In contrast, years with particularly high publication output, such as 2019–2022, reflected a rapid expansion in research productivity. Importantly, the maintenance of elevated citation ratios during these high-output years suggests that increased publication volume was not accompanied by a decline in overall research influence. These results are summarized in Supplementary Table S1 in the supplementary material.

To more accurately forecast the future trends in the total number of publications, we conducted a polynomial regression analysis using the available data. The equation of the trend line is as follows: y = −7E-05x⁶ + 0.0053x⁵ − 0.1475x⁴ + 1.9451x³ − 9.5693x² + 41.88x − 14.84, with a goodness-of-fit coefficient of *R*² = 0.9997. The positive upward trend depicted by the fitted curve indicates that the field of OCD remains in a phase of vigorous development and is likely to continue attracting considerable attention from researchers in the future.

### Countries/regions analysis

Tracing the source countries and regions of the retrieved publications and organizing or visualizing the data can help identify the hotspots of academic geography in the target research field, as well as uncover collaborative relationships between different regions. As shown in Table 1, the United States is the leading center in the field of OCD, with 729 articles and 40,208 citations, significantly outpacing the second-ranked country. This demonstrates the United States’ high influence and substantial strength in this field.

**Table 1:** Top ten countries/regions in terms of publication volume, total link strength, and citations in the field of obsessive-compulsive disorder (OCD) from 2000 to 2024

Rank	Countries	Documents	Countries	Citations	Countries	Total Link Strength
1	USA	729	USA	40,208	USA	760
2	China	265	England	16,689	England	512
3	Germany	237	Germany	10,663	Netherlands	475
4	England	223	Netherlands	9238	Germany	448
5	Canada	161	Canada	7360	Canada	417
6	Netherlands	160	Australia	7150	Spain	411
7	Australia	124	China	7149	Sweden	385
8	Japan	124	Spain	6295	Brazil	368
9	South Korea	115	Japan	5461	South Africa	363
10	Spain	111	Brazil	5326	China	342

The United Kingdom, with 223 articles and 16,689 citations, exhibits a high level of recognition, while Germany, with 237 articles and 10,663 citations, showcases its strong comprehensive capabilities. China (265 publications, 7,149 citations) and Canada (161 publications, 7,360 citations) demonstrate relatively high levels of activity. The Total Link Strength is an important indicator of the closeness of cooperation between countries, reflecting to some extent a country’s activity in international scientific research collaboration. The United States, Canada, and the Netherlands, among others, have significant scientific research influence, resource advantages, and core positions in the field of OCD, attracting numerous countries for collaborative exchanges.

**Fig. 3:**
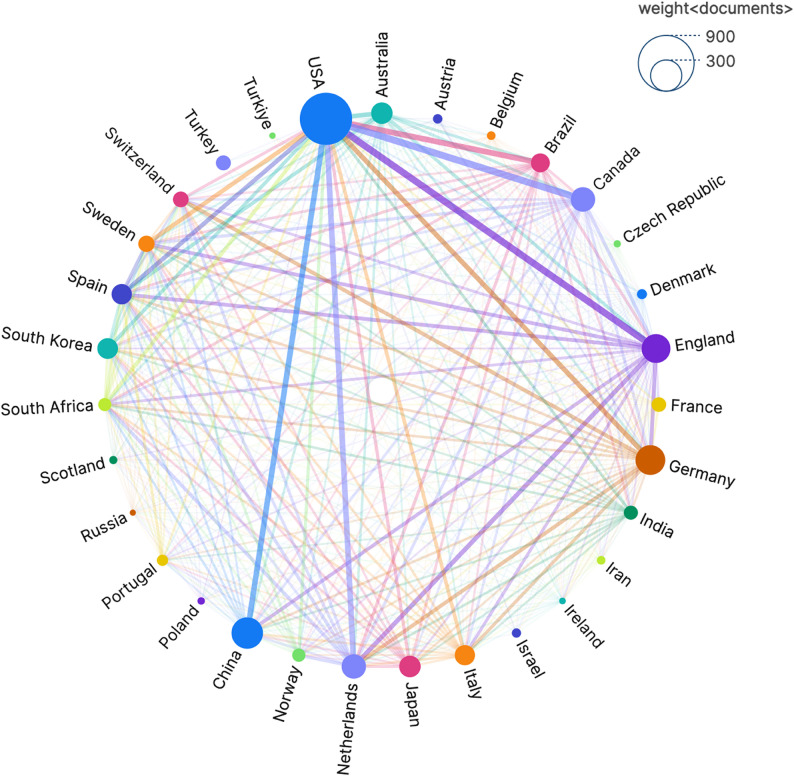
Global collaborative network analysis of OCD research from 2000 to 2024. A visual depiction of the research contributions by nation in the field of OCD, where nodes represent individual countries and connecting lines indicate collaborative relationships. Line width reflects the closeness and extent of cooperation. Node size represents the volume of publications from each country

To more intuitively illustrate the academic collaborations between active countries and regions within the field, we employed VOSviewer to visualize their scholarly exchange relationships and generated Fig. 3. In the figure, different nodes represent individual countries, collectively forming a network of academic exchanges. The connecting lines between nodes symbolize collaborative relationships between the respective countries or regions, with wider lines indicating closer and more extensive cooperation.

Judging from the number and width of the lines emanating from each node, the United States is the most active and influential country in the field of OCD, maintaining close and in-depth academic connections with the United Kingdom, Canada, Brazil, the Netherlands, and China, among others. This may be attributed to the fact that these countries share similar or complementary research themes within the field, and mutual exchanges can propel the advancement of research. The large number of nodes forming the network and the intricate web of connections within it suggest that the field of OCD is still in a period of vigorous development, attracting researchers from numerous countries and regions to engage in research activities.

### Author analysis

The data in Table 2 cover the top ten authors in the field of OCD in terms of publication output and co-citation frequency, as well as their respective countries/regions, institutions, and total link strength. Jun Soo Kwon from South Korea ranks first in publication output with 52 articles, indicating his high activity in the field and likely positioning him as a core researcher in OCD.

**Table 2:** Ranking of the top ten major authors in terms of documents and co-citations in the field of obsessive-compulsive disorder (OCD) from 2000 to 2024

Rank	Author	Documents	Total link strength	Countries/regions	Institution	Author	co-citations	Total link strength	Countries/regions	Institution
1	Kwon, Jun Soo	52	133	South Korea	Hanyang University Hospital	Goodman, Wk	939	15,620	USA	Baylor College of Medicine
2	Van Den Heuvel, Odile A.	43	162	Netherlands	Vrije Universiteit Amsterdam	Saxena, S	813	19,768	USA	University of California San Diego
3	Mataix-Cols, David	39	77	Spain	Karolinska Institutet	Rauch, Sl	729	16,501	USA	Harvard Medical School
4	Veltman, Dick J.	31	82	Netherlands	University of Amsterdam	Mataix-Cols, D	696	16,217	Spain	Karolinska Institutet
5	Gong, Qiyong	28	100	China	West China Hospital of Sichuan University	Menzies, L	580	13,003	England	Great Ormond Street Hospital for Children NHS Foundation Trust
6	Rauch, Scott L.	28	22	USA	Harvard Medical School	Van Den Heuvel, Oa	506	11,795	Netherlands	Vrije Universiteit Amsterdam
7	Atmaca, Murad	25	19	Turkey	Firat University	Chamberlain, Sr	502	12,357	England	University of Southampton
8	Harrison, Ben J.	25	94	Australia	University of Melbourne	Baxter, Lr	443	11,101	Canada	Novatox
9	Hoexter, Marcelo Queiroz	25	121	Brazil	Faculdade de Medicina da Universidade de São Paulo	Szeszko, Pr	430	10,606	USA	Feinstein Institute for Medical Research
10	Miguel, Euripedes Constantino	25	88	Brazil	Faculdade de Medicina da Universidade de São Paulo	Rosenberg, Dr	429	10,329	USA	Wayne State University

The second-ranked author in terms of publication output is Odile A. Van Den Heuvel from the Netherlands, with 43 publications. Notably, she also has the highest total link strength (162), suggesting her research has extensive international influence.

Two authors from Brazil, Marcelo Queiroz Hoexter and Euripedes Constantino Miguel, have relatively fewer publications (25), but their high total link strength indicates active international collaboration. This may suggest that their affiliated institution, Faculdade de Medicina da Universidade de São Paulo, is an emerging research center in the field of OCD, enhancing its research impact through international cooperation. The co-citation frequency of authors reflects the importance and relevance of their research within the field. Authors such as Wk Goodman (939 citations), S Saxena (813 citations), and Sl Rauch (729 citations) have significant influence in OCD research. These authors also have high total link strength, likely due to their affiliations with top-tier institutions known for their international collaboration opportunities, such as Baylor College of Medicine and the University of California San Diego.

**Fig. 4:**
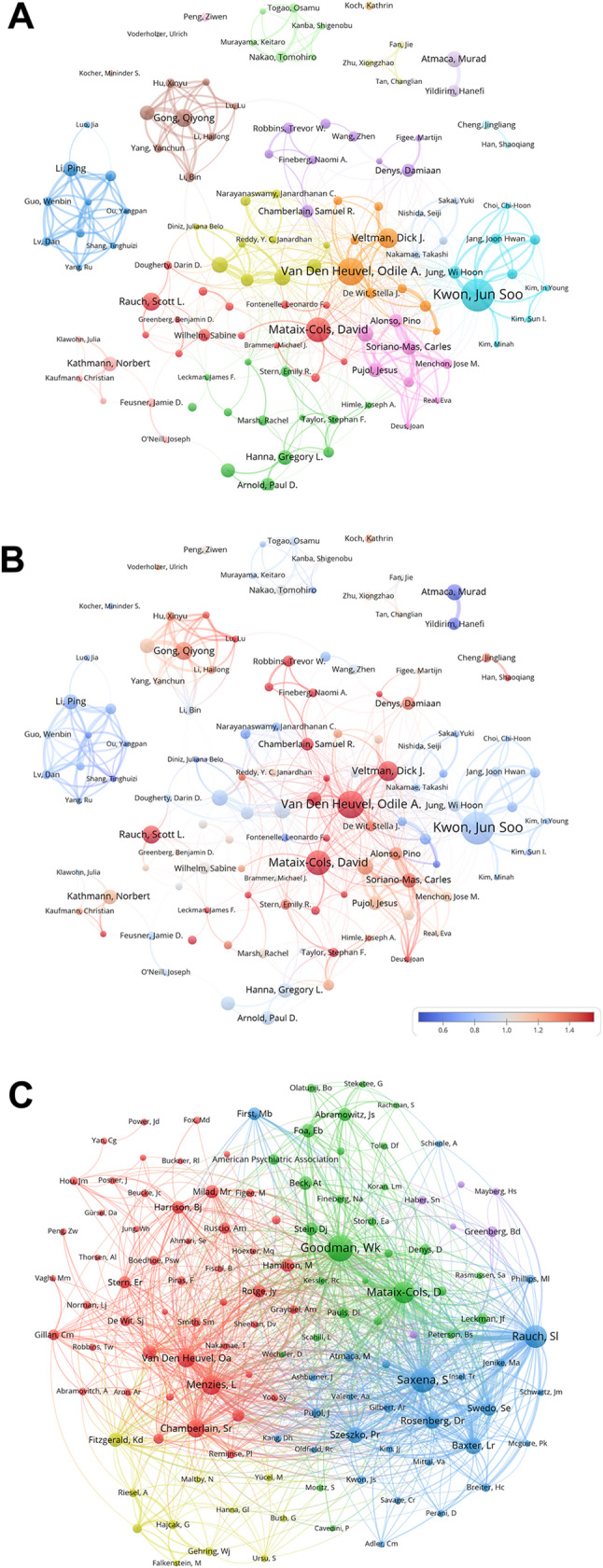
Network visualization of author collaboration and citation relationships in the field of OCD from 2000 to 2024. (**A**) This visualization provides a clear representation of collaborative authorship within OCD research. Authors are organized into distinct clusters based on their academic affiliations, with those sharing stronger scholarly ties typically positioned closer together. (**B**) The diagram illustrates the intensity of scholarly interactions using a color gradient, where blue indicates authors with fewer connections, and red denotes those with a denser network of relationships. (**C**) Authors are divided into four clusters based on their co-citation relationships, with node size corresponding to co-citation frequency. Larger nodes signify authors with higher co-citation frequencies

By analyzing the collaborative relationships among authors within the field, we can identify core authors and teams, uncover interdisciplinary and inter-institutional collaboration trends, and detect emerging research areas and hot topics, all of which contribute to promoting cross-disciplinary innovation. To this end, we analyzed and integrated the raw data on collaborative exchanges among authors in the field of OCD and generated Fig. 4.

In Fig. 4A, the co-occurrence network represents important authors as nodes, with larger nodes indicating higher publication output. Authors with close academic ties are clustered together in the same color, and the lines connecting nodes signify collaborative relationships between the corresponding authors. Several major clusters radiate from central nodes within the figure.

For instance, the light blue cluster on the right side, centered around the most prolific author, Jun Soo Kwon, primarily comprises authors from Seoul National University. The pink cluster, represented by Carles Soriano-Mas, includes authors mainly from the University of Barcelona, who have significant publication output. The central orange cluster consists mostly of authors from the University of Amsterdam in the Netherlands, with key figures such as Odile A. Van Den Heuvel and Dick J. Veltman. The purple cluster contains authors predominantly from the United Kingdom. The green cluster at the bottom includes authors largely affiliated with the University of Michigan or its surrounding institutions, with notable authors Gregory L. Hanna and Paul D. Arnold also belonging to the OCD Foundation Genetics Consortium (OCDF GC).

The light red cluster in the lower left corner is mostly composed of authors from the Humboldt University of Berlin in Germany, with representative authors such as Norbert Kathmann, Christian Kaufmann, and Julia Klawohn. The red cluster, represented by Scott L. Rauch and Sabine Wilhelm, includes authors mainly from Harvard University, Harvard Medical School, and Brown University. However, authors with multiple institutional affiliations, such as David Mataix-Cols and Leonardo F. Fontenelle, also have close collaborations with this cluster.

The blue and brown clusters on the left side are predominantly composed of authors from China. The blue cluster features a more diverse range of institutional affiliations, with authors such as Wenbin Guo, Yangpan Ou, and Dan Lv from Central South University, and Tinghuizi Shang from Qiqihar Medical University. The brown cluster mainly includes authors from institutions in the southwestern region of China, particularly Sichuan University and its affiliated West China Hospital of Sichuan University, with representative authors such as Qiyong Gong, Yanchun Yang, and Xinyu Hu.

As shown in Fig. 4B, European authors represented by orange, red, and pink clusters often have close and extensive academic exchanges, which may explain the significant publication output of many authors in this region. Authors with multiple institutional affiliations tend to have noticeable collaborative relationships across different clusters, thereby exerting considerable influence within the field.

Analyzing the co-citation relationships among authors can identify core authors within the field, assess their academic influence, and reveal potential correlations between their research topics. To this end, we processed the relevant data and generated Fig. 4C. In the figure, authors are represented by nodes and are broadly categorized into four colors (red, green, blue, and yellow) based on the relevance of their research areas. A connecting line between two nodes indicates a co-citation relationship between the corresponding authors, with thicker lines representing stronger co-citation intensity.

It can be observed that authors in the green cluster, represented by WK Goodman, D Mataix-Cols, and DJ Stein, primarily focus on research areas such as OCD, anxiety disorders, behavioral addictions, and internet use issues within the field of psychiatry. Their research encompasses fundamental neurobiological mechanisms, clinical treatments, and interdisciplinary methodologies, enabling in-depth collaboration and knowledge sharing across multiple levels. Authors in the blue cluster have significant research contributions in the fields of psychiatry, neuroscience and neurology, pharmacology and pharmacy, and psychology. Similar to the green cluster, their work covers fundamental neurobiological mechanisms, clinical treatments, and interdisciplinary approaches, facilitating extensive collaboration and knowledge integration. Representative authors in this cluster include S Saxena, Sl Rauch, and Pr Szekzko.

On the left side of the figure, the red cluster comprises researchers whose work overlaps significantly in the fields of psychiatry, neuroscience and neurology, psychology, radiology, nuclear medicine, and medical imaging. Their research directions share substantial commonalities and synergies. Representative authors in this cluster include Odile A. Van Den Heuvel, L Menzies, and Sr Chamberlain. In the upper right corner of the figure, the purple nodes represent authors who have made notable contributions to the field of psychiatry, particularly in the study of neuro-psychiatric disorders such as OCD, anxiety disorders, and Parkinson’s disease. Although the research directions of these authors may not be universally prevalent, they share similarities or complementary aspects with those in the green or blue clusters, resulting in their distribution within these two clusters.

### Institution analysis

Table 3 presents the top 10 institutions in the field of OCD in terms of publication output and citation frequency. It is evident that half of the top 10 institutions in terms of publication output are from the United States, and most of these institutions also have high citation frequencies. Representative institutions include Harvard University (67 publications, 5,611 citations), Massachusetts General Hospital (54 publications, 4,724 citations), and Yale University (51 publications, 6,435 citations). These figures further confirm the substantial research capacity and significant academic influence of the United States in this field.

**Table 3:** Ranking of the top ten major institutions of obsessive-compulsive disorder (OCD) from 2000 to 2024

Rank	Institution	Publications	Original Country	Institution	Citations	Original Country
1	King’s College London	70	England	Yale University	6435	USA
2	Harvard University	67	USA	King’s College London	5727	England
3	University of Toronto	66	Canada	Harvard University	5611	USA
4	Seoul National University	59	South Korea	University of Cambridge	5340	England
5	University of Cambridge	56	England	Massachusetts General Hospital	4724	USA
6	Vrije Universiteit Amsterdam	56	Netherlands	Vrije Universiteit Amsterdam	3296	Netherlands
7	Massachusetts General Hospital	54	USA	University of Pittsburgh	3204	USA
8	University of Michigan	53	USA	University of Toronto	2976	Canada
9	Harvard Medical School	51	USA	Seoul National University	2905	South Korea
10	Yale University	51	USA	University of Sao Paulo	2769	Brazil

The research strength of the United Kingdom in the field of OCD is also notable. The institution with the highest publication output is King’s College London from the UK (70 publications), while the second and fourth highest citation frequencies are held by King’s College London (5,727 citations) and the University of Cambridge (5,340 citations), respectively.

It is also worth highlighting the University of São Paulo from Brazil, which, although not prominent in terms of publication output, has a relatively high citation frequency. This indicates that the research conducted by this institution in the field of OCD is highly recognized and has made significant contributions.

**Fig. 5:**
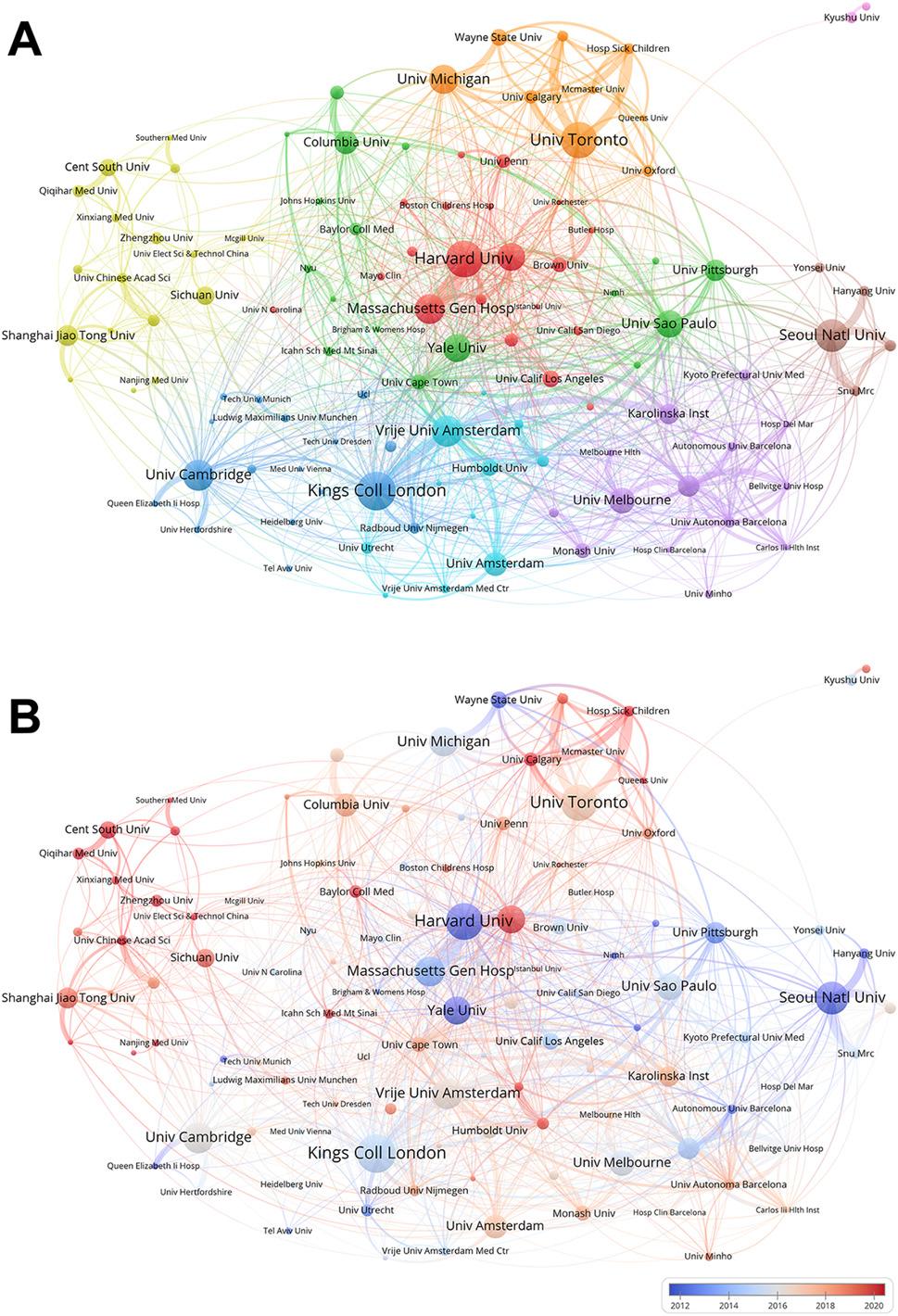
Institutional collaborative network mapping of OCD research from 2000 to 2024. (**A**) The graph displays the co-occurrence of research institutions, where the size of a node is indicative of the frequency of collaborative occurrences and the lines signify the relationships of co-occurrence. The larger the node, the more frequently the institution is involved in collaborations, and the interconnections denote the instances where they appear together. (**B**) The figure illustrates the timing of academic exchanges between institutions, where nodes colored closer to blue indicate earlier interactions, and those closer to red signify recent and intensive collaborative exchanges

Figure 5 presents the collaboration network of key institutions in the field of OCD, where nodes represent institutions. In Fig. 5A, institutions with close academic ties are grouped into clusters of the same color, and the lines connecting nodes illustrate their collaborative relationships. A detailed analysis reveals that these collaborative relationships also exhibit strong geographical attributes.

Institutions within the orange cluster are primarily located in the United States and Canada, with representative institutions including the University of Michigan, University of Toronto, and Queen’s University. Notably, the University of Oxford from the United Kingdom is also included in the orange cluster. This may be attributed to the broader geographical reach, which facilitates the involvement of a wider range of participants and research subjects, thereby enhancing the generalizability and representativeness of OCD research.

The red cluster predominantly comprises institutions from the United States, with the exception of Istanbul University from Turkey. Representative institutions include the University of Pennsylvania, University of California, San Diego, and Harvard University. These institutions have a long-standing tradition of extensive academic connections and collaborations. For example, institutions such as Harvard University, Massachusetts General Hospital, and Brown University have consistently maintained close collaborative relationships in the fields of neuroscience and psychiatry. This long-standing tradition of collaboration provides a solid foundation for OCD research.

Institutions within the green cluster are mostly leading medical and research institutions in their respective countries and regions. They possess distinct strengths in fields such as neuroscience, psychiatry, psychology, neuroimaging, and biomedical engineering. Through collaboration, these institutions can achieve interdisciplinary complementarity, thereby advancing research in the field of OCD. The Icahn School of Medicine at Mount Sinai is a leading institution in the field of OCD research, particularly in combining computational psychiatry with deep brain stimulation (DBS) techniques to investigate the neurobiological mechanisms underlying OCD. This innovative approach leverages advanced computational models and neuroimaging techniques to better understand the neural circuits involved in OCD and develop more effective treatments.

Collaborations between institutions such as Yale University and the University of Pittsburgh are also crucial for advancing OCD research. These institutions, often supported by funding from the National Institutes of Health, utilize their strong clinical and research foundations to conduct cutting-edge studies. For example, Yale University has a long history of groundbreaking research in OCD, including the development of the Yale-Brown Obsessive Compulsive Scale (Y-BOCS) and the first clinical trials demonstrating the efficacy of SSRIs for OCD treatment. The University of Pittsburgh is similarly active in this field, contributing to both clinical and neurobiological research.

In the green cluster, institutions such as the University of São Paulo, the University of Cape Town, and Minh University are actively collaborating with leading U.S. institutions like the University of Pittsburgh and Yale University. These collaborations help expand their academic influence and research networks by leveraging shared research interests and resources.

The blue cluster includes top-tier research institutions from the UK and Germany, such as King’s College London, the University of Cambridge, and the Technical University of Munich. These institutions are renowned for their contributions to neuroscience, psychiatry, and related fields. The inclusion of institutions like the Medical University of Vienna and Radboud University Nijmegen further enriches the collaborative network with diverse European perspectives. Tel Aviv University, located at the crossroads of Asia, Europe, and Africa, brings additional international diversity to this cluster. The purple cluster in the lower left corner of the figure encompasses institutions from multiple countries, including Hospital del Mar from Spain, University of Melbourne from Australia, Kyoto Prefectural University of Medicine from Japan, and Karolinska Institutet from Sweden. The diverse regional resources of these institutions enable a more comprehensive understanding of OCD as a global health issue and facilitate the development of more effective treatment approaches.

The light blue cluster primarily consists of institutions from the Netherlands, such as Vrije Universiteit Amsterdam and University of Amsterdam. The only representative institution not from the Netherlands is Humboldt-Universität zu Berlin from Germany. This institution actively engages in student exchanges with several Dutch universities through programs like Erasmus+, which, combined with the linguistic similarities between the two countries, greatly facilitates close collaboration.

The yellow cluster on the left side of the figure is predominantly composed of Chinese institutions, while the brown cluster on the right side consists exclusively of South Korean institutions. These clusters reflect a strong geographical limitation in their collaborative relationships. As shown in Fig. 5B, which presents the temporal dimension of institutional collaboration, cooperation among Chinese institutions began relatively late. This may be attributed to the later development of the field of OCD in China. Deep collaboration between high-level institutions facilitates resource sharing and complementary capabilities, which is conducive to achieving breakthroughs in a shorter period of time. In contrast, institutions with more significant publication output in the figure generally initiated collaboration earlier, laying a solid foundation for research in the field of OCD. Representative institutions include Harvard University, Seoul National University, and Yale University, among others.

### Journal analysis

Table 4 was compiled by tracing the source journals of articles related to OCD on the Web of Science and analyzing the information. This table presents the top 10 journals with the highest number of publications and citations, as well as their impact factors (IF) and Journal Citation Reports (JCR) quartiles. According to the table, *Psychiatry Research-Neuroimaging* is the journal with the highest number of publications, having published 82 articles. Other journals with over 50 publications include *Biological Psychiatry* (58 articles), *Frontiers in Psychiatry* (58 articles), and *Journal of Affective Disorders* (55 articles). In terms of citations, the journals with over 4,000 citations are *Archives of General Psychiatry* (5,523 citations), *Biological Psychiatry* (5,435 citations), *Neuroimage* (4,827 citations), and *American Journal of Psychiatry* (4,236 citations).

**Table 4:** Ranking of the top ten major journals of obsessive-compulsive disorder (OCD) from 2000 to 2024

Rank	Journal	Publications	IF(JCR2023)	JCR quatile	Co-Cited-Journal	Citations	IF(JCR2023)	JCR quatile
1	Psychiatry Research-Neuroimaging	82	2.1	Q3	Archives of General Psychiatry	5523	14.480(2014)	Q1(2014)
2	Biological Psychiatry	58	9.6	Q1	Biological Psychiatry	5435	9.6	Q1
3	Frontiers in Psychiatry	58	3.2	Q2	Neuroimage	4827	4.7	Q1
4	Journal of Affective Disorders	55	4.9	Q1	American Journal of Psychiatry	4236	15.1	Q1
5	Neuroimage-Clinical	44	3.4	Q2	Psychiatry Research - Neuroimaging	2209	2.1	Q3
6	Human Brain Mapping	43	3.5	Q1	Human Brain Mapping	1806	3.5	Q1
7	Journal of Psychiatric Research	39	3.7	Q1	Journal of Neuroscience	1691	4.4	Q1
8	Progress In Neuro-Psychopharmacology & Biological Psychiatry	37	5.3	Q1	Neuroscience and Biobehavioral Reviews	1622	7.6	Q1
9	Psychological Medicine	37	5.9	Q1	Neuropsychopharmacology	1618	6.6	Q1
10	Journal of Obsessive-Compulsive and Related Disorders	36	1.9	Q3	Behaviour Research and Therapy	1576	4.2	Q1

Considering the JCR quartile and IF of the journals provides a preliminary assessment of their influence within the field. High-impact journals typically cover broad research areas such as psychiatry and neuroscience, thereby attracting more interdisciplinary research submissions and citations. Representative journals in this regard include *Archives of General Psychiatry* and *Biological Psychiatry*. It is also worth noting that journals such as the Journal of *Obsessive-Compulsive and Related Disorders*, although having relatively lower citation frequencies, demonstrate a higher degree of focus on OCD research and thus merit closer attention.

**Fig. 6:**
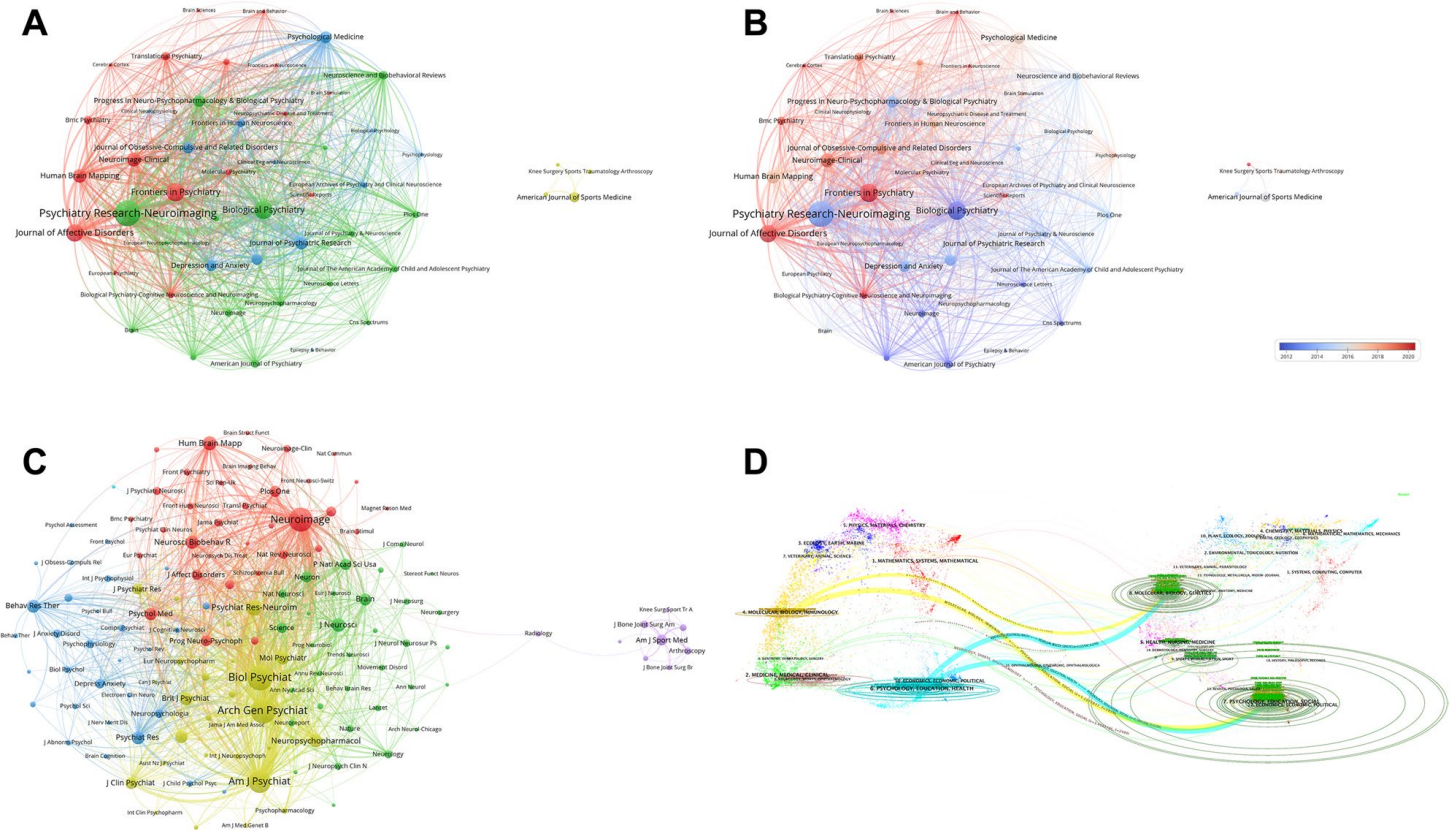
Network visualization of journal publication volume, collaboration, and citation relationships in the field of OCD from 2000 to 2024. (**A**) Co-occurrence relationships among journals in OCD research. Nodes represent journals and are categorized by color into three main clusters. Journals not belonging to these clusters are shown in yellow, indicating distinct research directions. Dense connections between nodes highlight strong collaborative relationships among key journals. (**B**) The graph traces the timeline of journal cooperation within the OCD field, with blue nodes indicating journals that have been active from the outset and red nodes highlighting more recent entrants to the domain. (**C**) This graph delineates co-citation connections among scholarly journals, where the size of the nodes corresponds to the frequency of co-occurrences, and the edges signify the relationships between journals. The magnitude of each node is proportional to the journal’s prominence and influence within the scholarly network. (**D**) The dual map visualizes journals related to OCD research, with clusters of citing journals on the left and cited journals on the right. Citation relationships are represented by colored trajectories connecting them

Figure 6A and B present the collaboration networks of key journals in the field of OCD. Figure 6A illustrates the co-occurrence relationships among these journals, while Fig. 6B further refines Fig. 6A based on the timing of journal publications.

In Fig. 6A, the nodes representing different journals are primarily divided into three colors: red, blue, and green. Journals that are not part of the main clusters are represented by yellow nodes, likely due to their distinct research directions compared to other journals. The connections between nodes indicate collaborative relationships among the journals. The dense connections and the lack of clear distinctions between clusters suggest that key journals in the OCD field have deep collaborative relationships. The green cluster features prominent nodes such as *Psychiatry Research-Neuroimaging* and *Biological Psychiatry*. The red cluster is centered around journals like *Journal of Affective Disorders*, *Frontiers in Psychiatry*, and *Neuroimaging-Clinical*, forming a dispersed cluster. The blue cluster includes journals such as *Psychological Medicine*, *Journal of Psychiatric Research*, and *Depression and Anxiety*. Figure 6B reveals that journals in the red cluster generally began publishing articles on OCD relatively recently, suggesting that they have only recently started to focus on OCD research.

Analyzing the co-citation relationships among journals can assist researchers in better understanding the underlying reasons for collaboration between journals. To this end, we conducted an analysis of this data and generated Fig. 6C. In the figure, nodes represent different journals, with larger diameters indicating higher co-citation frequencies. The density of connections between nodes signifies the degree of co-citation intensity between the respective journals. The nodes are broadly categorized into four colors: red, yellow, blue, and green. Journals within the yellow cluster exhibit high co-citation frequencies, such as *Biological Psychiatry*, *Archives of General Psychiatry*, and *American Journal of Psychiatry*. The red cluster includes journals like *Neuroimage*, *Human Brain Mapping*, and *Neuroscience and Biobehavioral Reviews*, which also demonstrate high co-citation frequencies. The blue cluster on the left side of the figure is represented by journals such as *Behavior Research and Therapy*, *Psychiatry Research*, and *Depression and Anxiety*. Journals in these clusters all focus on neuroscience, psychology, and psychiatry. However, those in the yellow cluster are more concentrated on the biological basis of mental disorders, diagnosis, treatment, and epidemiological research, while journals in the blue cluster focus on the diagnosis, treatment, and fundamental research of mental health disorders. The green cluster on the right side of the figure comprises journals that specialize in neuroscience-related research, with representative journals including *Journal of Neuroscience*, *Brain*, and *Neuron*.

Figure 6D presents a dual-mapping overlay of citation relationships and changes in research focus among key journals in the field of OCD. After categorizing the research directions of the journals into different clusters, arrows are used to indicate the citation relationships between them. The direction of the arrows points from the citing journals to the cited journals. There are four prominent bands in the figure, with two bands emanating from clusters numbered 4 and 6, respectively, pointing towards clusters numbered 8 and 7. A detailed analysis revealed that the band pointing towards cluster 7 is more pronounced than the other band of the same color. This indicates that journals focusing on Molecular, Biology, and Immunology, as well as those on Psychology, Education, and Health, are more likely to cite journals that concentrate on Psychology, Education, and Social, rather than those focusing on Molecular, Biology, and Genetics.

### Keywords analysis

Keywords, as concise summaries of a paper’s research direction or content, can often reveal developmental trends and cutting-edge research foci within a specific field through co-occurrence analysis. The 20 keywords listed in Table 5 are the most frequently occurring terms in the field of OCD, and their associations and significance within the field can be comprehensively analyzed in conjunction with their total link strength.

**Table 5:** Ranking of the top twenty major keywords of obsessive-compulsive disorder (OCD) from 2000 to 2024

Rank	Keyword	Occurrences	Total link strength	Rank	Keyword	Occurrences	Total link strength
1	obsessive-compulsive disorder	1234	2399	11	event-related potentials	53	126
2	fmri	230	630	12	depression	51	163
3	mri	202	449	13	knee	46	100
4	neuroimaging	142	412	14	error-related negativity	45	112
5	deep brain stimulation	86	216	15	schizophrenia	45	139
6	diffusion tensor imaging	83	214	16	orbitofrontal cortex	43	132
7	cognitive behavioral therapy	79	200	17	meta-analysis	39	112
8	eeg	78	169	18	voxel-based morphometry	38	91
9	functional connectivity	72	170	19	exposure and response prevention	37	74
10	anxiety	55	157	20	anxiety disorder	36	105

The term “obsessive-compulsive disorder,” as the core keyword of the field, leads all other terms with 1,234 occurrences and a total link strength of 2,399, thereby establishing a keyword network that interconnects the entire field. Other keywords with over 100 occurrences include “fmri” (230 times), “mri” (202 times), and “neuroimaging” (142 times). These terms also exhibit high total link strength, indicating that they reflect core research themes within the field. Their prominence suggests that they are likely to shape future research trends and provide guidance for the establishment of research directions.

**Fig. 7:**
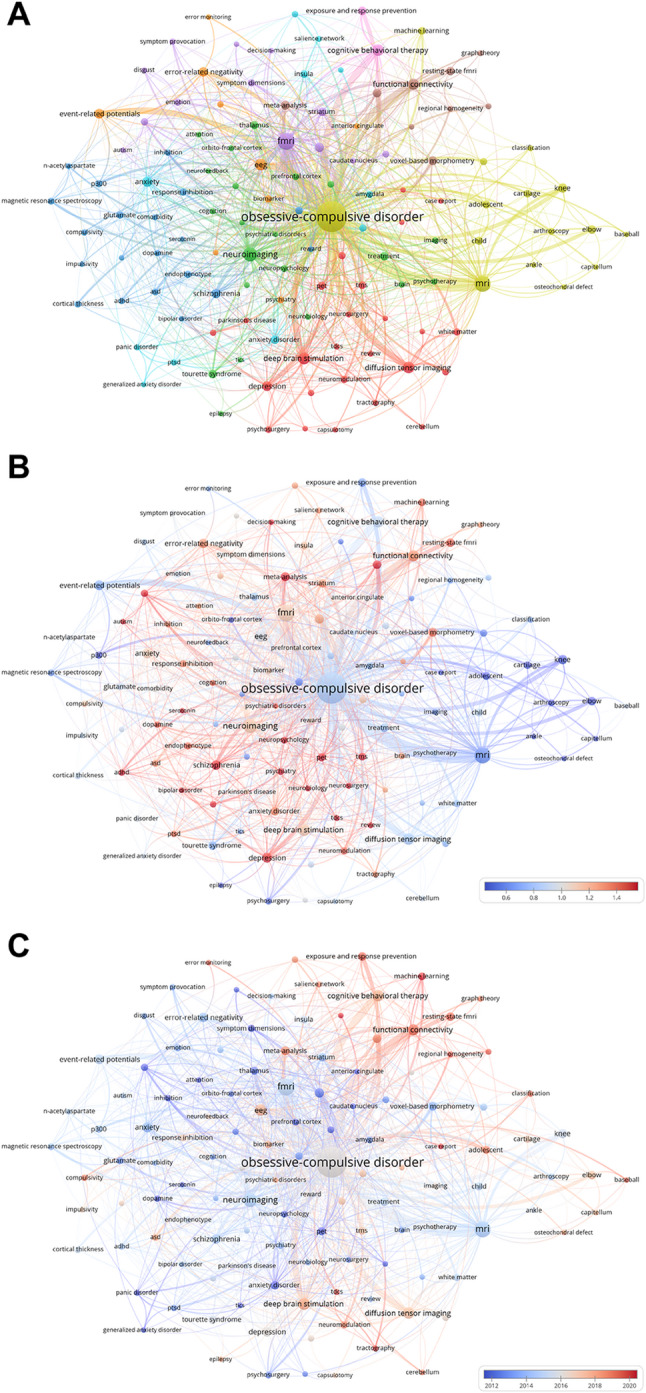
Visualization of keyword co-occurrence in OCD research spanning from 2000 to 2024. (**A**) The keyword map illustrates the interconnections among keywords in the OCD research domain. Nodes, differentiated by color, signify distinct clusters of keywords. Node size corresponds to co-occurrence frequency, while connections between nodes represent associative relationships among keywords. (**B**) The figure illustrates the correlation between the recent scholarly contributions of each keyword to OCD research and its overall body of work. Red shades indicate an increase in a keyword’s influence, while blue signifies decreasing attention within the field. The color scale reflects the proportional representation of keywords over the past four-year period, highlighting terms that have had a substantial impact or experienced a decline in their role within this research area. (**C**) A heat map, overlaid on the graph based on Fig. 7A, uses varying color shades to depict the recent attention levels accorded to different keywords

Figure 7A illustrates the co-occurrence relationships among keywords. Combined with the keyword co-occurrence strength map and the keyword occurrence timeline map in Fig. 6B and C, it can help researchers quickly understand the connections among hot keywords and the development trends of research hotspots within the OCD field. In Fig. 7A, the keyword with the largest node diameter is “obsessive-compulsive disorder,” which also has the richest connections, making it the core keyword in the OCD field. The largest cluster in the figure is the red cluster at the bottom, where the keywords are more related to research in neuroimaging, modulatory techniques, and surgery, with representative terms including “diffusion tensor imaging,” “capsulotomy,” and “deep brain stimulation.”

The purple, orange, and green clusters are interwoven with each other. The keywords within these clusters have developed complex co-occurrence relationships due to their strong association with the field of neuroscience. However, the keywords in the purple cluster are more focused on research related to autism spectrum disorders and other neurodevelopmental disorders, with representative terms including “fMRI”, “symptom dimensions”, and “symptom provocation”. The orange cluster can be further categorized into cognitive neuroscience, with representative terms such as “error-related negativity”, “event-related potentials”, and “error monitoring”. The light blue cluster centers on anxiety disorder research, with representative terms like “generalized anxiety disorder”, “anxiety disorder”, and “panic disorder”. The keywords in yellow cluster are mostly related to children and adolescent. The blue cluster contains a variety of popular keywords spanning neuroscience, psychiatry, and psychology, particularly focusing on neurodevelopmental disorders and neuropsychiatric diseases, with representative terms such as “compulsivity”, “bipolar disorder”, and “schizophrenia”.

Keywords in the green cluster are scattered across other clusters, indicating broader connections with other clusters. These keywords pertain to research in cognitive neuroscience, clinical neuroscience, and neurodegenerative diseases, with representative terms including “thalamus,” “neuroimaging,” and “neuropsychology.” The red cluster encompasses keywords related to neuroimaging, modulatory techniques, and surgery, with representative terms such as “diffusion tensor imaging,” “capsulotomy,” and “deep brain stimulation.” The brown cluster in the upper right corner focuses on brain function and structure from a neuroimaging perspective, with representative terms including “voxel-based morphometry,” “regional homogeneity,” and “resting-state fMRI.” The pink cluster contains two popular keywords related to the psychological treatment of OCD, such as “cognitive behavioral therapy” and “exposure and response prevention.”

Further analysis in conjunction with Fig. 6B and C reveals that keywords such as “schizophrenia,” “ADHD,” and “depression” in the lower left corner of Fig. 7A have high popularity. In contrast, keywords within the brown cluster have garnered significant attention in the OCD field only in recent years, representing the current frontier of OCD research from psychological and neuroscientific perspectives.

**Fig. 8:**
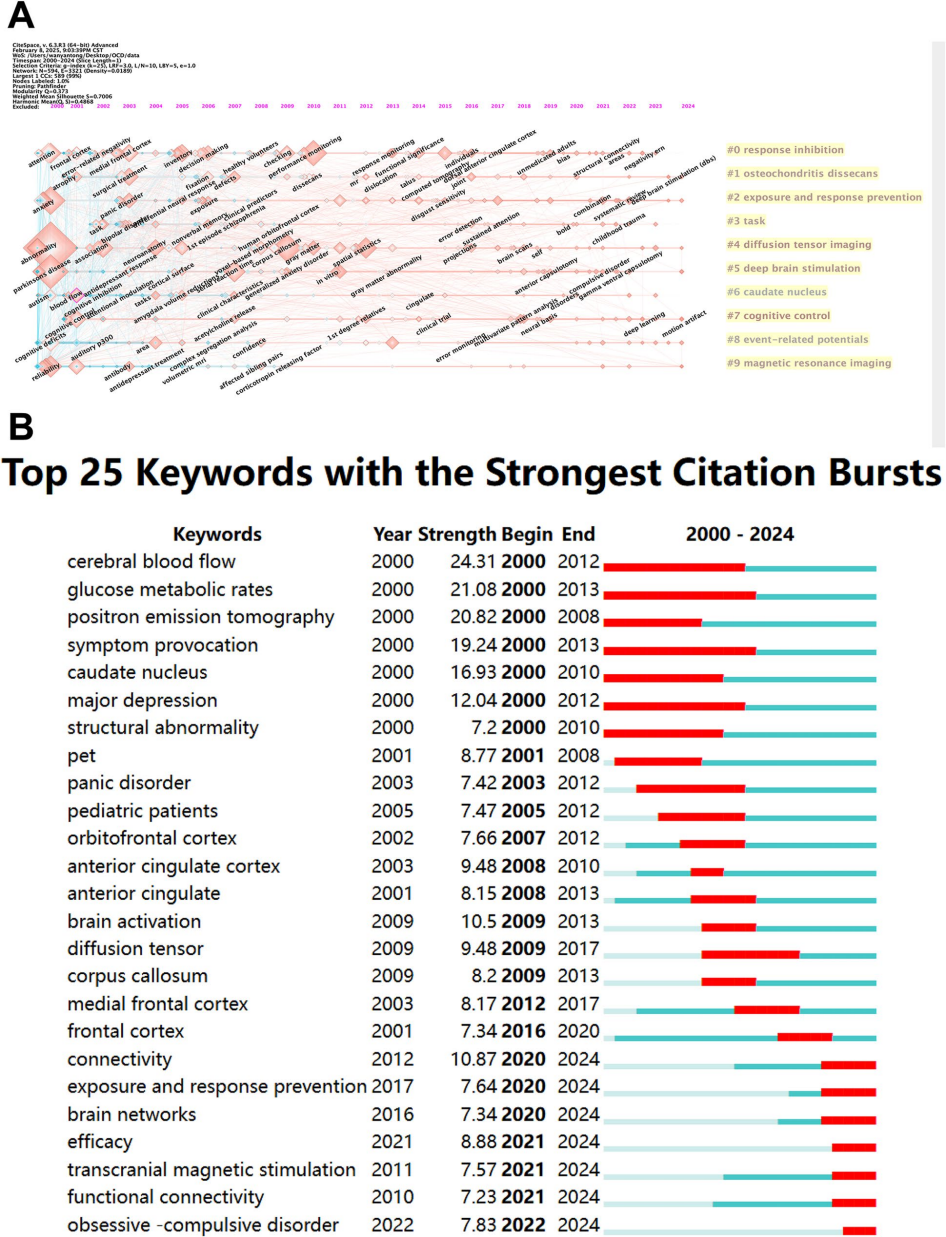
Analysis of keyword citation trends in OCD research from 2000 to 2024. (**A**) Developmental trends of the top 10 keyword clusters over time. Each horizontal line represents a keyword cluster, with node size proportional to co-citation frequency. Cluster #0 is the largest. (**B**) The diagram highlights 25 keywords marked by red spikes along the timeline, indicating significant bursts in citations

We categorized the popular keywords into different clusters and compared the popularity and developmental trends of the top 10 clusters both horizontally and vertically across the timeline, with the results presented in Fig. 8A. In the figure, Cluster #0 is the largest cluster. Each horizontal line represents a keyword cluster, and the size of the nodes on the line is proportional to the co-citation frequency. For example, in Cluster #0 “response inhibition,” the earliest emerging popular keyword is “attention.” Other clusters that also attracted the attention of researchers relatively early include Cluster #2 “exposure and response prevention,” Cluster #4 “diffusion tensor imaging,” Cluster #5 “deep brain stimulation,” and Cluster #9 “magnetic resonance imaging.” The high co-cited keywords in these clusters are “anxiety,” “abnormality,” “Parkinson’s disease,” and “reliability,” respectively. It can be observed that in recent years, no obvious new popular keywords have emerged in the field. This may be because research on OCD has reached a certain level of maturity. Compared to chasing new hotspots, researchers are more inclined to continue focusing on specific core issues.

Figure 8B illustrates the citation bursts of the 25 most frequently occurring keywords in the field of OCD. The longest-lasting bursts were observed for “glucose metabolic rates” and “symptom provocation,” with their popularity persisting from 2000 to 2013. The second-longest bursts were for “cerebral blood flow” and “major depression,” which remained popular for 12 years (2000–2012). In terms of burst intensity, the keywords exceeding a value of 20 include “cerebral blood flow” (24.31), “glucose metabolic rates” (21.08), and “positron emission tomography” (20.82). These high-intensity bursts suggest that the associated research topics may have experienced key breakthroughs, attracting significant attention from researchers within a short period. Some keywords with later emergence dates have lower burst intensities but have maintained their popularity to the present day. This indicates that these keywords may represent emerging research themes in the field, warranting further exploration.

### Highly cited references analysis

The citation frequency of an article can intuitively reflect its influence within a specific field, and the research directions of highly cited articles often represent popular research themes in that field. Therefore, we compiled a list of the 15 most cited articles in the field of OCD, along with their authors, source journals, and publication years, as presented in Table 6. This information is intended to provide a rapid overview of the primary research directions in the field and to track emerging hotspots.

**Table 6:** Ranking of the top fifteen major highly cited references of obsessive-compulsive disorder (OCD) from 2000 to 2024

Rank	Author		Source Title	Cited	Year	Category	DOI
1	van Veen, V; Carter, CS	The anterior cingulate as a conflict monitor: fMRI and ERP studies	Physiology and Behavior	1031	2002	Article	10.1016/S0031-9384(02)00930-7
2	Goodkind, M; Eickhoff, SB; Oathes, DJ et al.	Identification of a Common Neurobiological Substrate for Mental Illness	Jama Psychiatry	941	2015	Article	10.1001/jamapsychiatry.2014.2206
3	Menzies, L; Chamberlain, SR; Laird, AR et al.	Integrating evidence from neuroimaging and neuropsychological studies of obsessive-compulsive disorder: The orbitofronto-striatal model revisited	Neuroscience and Biobehavioral Reviews	874	2008	Review	10.1016/j.neubiorev.2007.09.005
4	Mataix-Cols, D; do Rosario-Campos, MC; Leckman, JF	A multidimensional model of obsessive-compulsive disorder	American Journal of Psychiatry	716	2005	Review	10.1176/appi.ajp.162.2.228
5	Radua, J; Mataix-Cols, D	Voxel-wise meta-analysis of grey matter changes in obsessive-compulsive disorder	British Journal of Psychiatry	692	2009	Review	10.1192/bjp.bp.108.055046
6	Mataix-Cols, D; Wooderson, S; Lawrence, N et al.	Distinct neural correlates of washing, checking, and hoarding symptom dimensions in obsessive-compulsive disorder	Archives of General Psychiatry	689	2004	Article	10.1001/archpsyc.61.6.564
7	Chamberlain, SR; Blackwell, AD; Fineberg, NA et al.	The neuropsychology of obsessive compulsive disorder: the importance of failures in cognitive and behavioural inhibition as candidate endophenotypic markers	Neuroscience and Biobehavioral Reviews	630	2005	Review	10.1016/j.neubiorev.2004.11.006
8	Milad, MR; Rauch, SL	Obsessive-compulsive disorder: beyond segregated cortico-striatal pathways	Trends in Cognitive Sciences	590	2012	Review	10.1016/j.tics.2011.11.003
9	Pauls, DL; Abramovitch, A; Rauch, SL et al.	Obsessive-compulsive disorder: an integrative genetic and neurobiological perspective	Nature Reviews Neuroscience	527	2014	Review	10.1038/nrn3746
10	Parkes, L; Fulcher, B; Yücel, M et al.	An evaluation of the efficacy, reliability, and sensitivity of motion correction strategies for resting-state functional MRI	Neuroimage	480	2018	Article	10.1016/j.neuroimage.2017.12.073
11	Fineberg, NA; Potenza, MN; Chamberlain, SR et al.	Probing Compulsive and Impulsive Behaviors, from Animal Models to Endophenotypes: A Narrative Review	Neuropsychopharmacology	473	2010	Review	10.1038/npp.2009.185
12	Bloch, MH; Landeros-Weisenberger, A; Rosario, MC et al.	Meta-Analysis of the Symptom Structure of Obsessive-Compulsive Disorder	American Journal of Psychiatry	468	2008	Review	10.1176/appi.ajp.2008.08020320
13	Newson, JJ; Thiagarajan, TC	EEG Frequency Bands in Psychiatric Disorders: A Review of Resting State Studies	Frontiers in Human Neuroscience	459	2019	Review	10.3389/fnhum.2018.00521
14	Harrison, BJ; Soriano-Mas, C; Pujol, J et al.	Altered Corticostriatal Functional Connectivity in Obsessive-compulsive Disorder	Archives of General Psychiatry	449	2009	Article	10.1001/archgenpsychiatry.2009.152
15	Fox, MD; Buckner, RL; Liu, HS et al.	Resting-state networks link invasive and noninvasive brain stimulation across diverse psychiatric and neurological diseases	Proceedings of the National Academy of Sciences of the United States of America	429	2014	Article	10.1073/pnas.1405003111

The most cited article is “The anterior cingulate as a conflict monitor: fMRI and ERP studies” [28] by van Veen, V; Carter, CS, published in *Physiology and Behavior* in 2002. This study primarily investigates the role of the anterior cingulate cortex (ACC) in conflict monitoring. Utilizing functional magnetic resonance imaging (fMRI) and event-related potentials (ERP), the authors found that the ACC plays a critical role in detecting cognitive conflict and modulating behavioral responses, supporting the theory of the ACC as a conflict monitor.

The second most cited article, with 941 citations, is “Identification of a Common Neurobiological Substrate for Mental Illness” [29] by Goodkind, M; Eickhoff, SB; Oathes, DJ; Jiang, Y; Chang, A; Jones-Hagata, LB; Ortega, BN; Zaiko, YV; Roach, EL; Korgaonkar, MS; Grieve, SM; Galatzer-Levy, I; Fox, PT; Etkin, A, published in *JAMA Psychiatry* in 2015. This study conducted a meta-analysis of structural neuroimaging studies across various mental illnesses, identifying common brain regions with gray matter reductions in patients with schizophrenia, bipolar disorder, depression, addiction, OCD, and anxiety. These regions, including the dorsal anterior cingulate cortex and bilateral insula, form a tightly connected network in healthy individuals, and gray matter reductions are associated with executive function impairments.

The third most cited article, with 874 citations, is a review titled “Integrating evidence from neuroimaging and neuropsychological studies [30]” by L Menzies; SR Chamberlain; AR Laird; SM Thelen; BJ Sahakian; ET Bullmore, published in *Neuroscience and Biobehavioral Reviews* in 2008. This review integrates evidence from neuroimaging and neuropsychological studies, re-examining the orbitofrontal-striatal model of OCD. It explores the neurofunctional abnormalities in the orbitofrontal cortex and striatal regions in OCD patients and their relationship with cognitive and behavioral performance.

The information in the table indicates that review articles are overrepresented among the highly cited papers. This may be attributed to the complexity and multidimensionality of the OCD field, where many fundamental theories remain incompletely established. Review articles can synthesize research findings from multiple dimensions, helping researchers to organize existing results and propose new theories, thereby being widely cited.

**Fig. 9:**
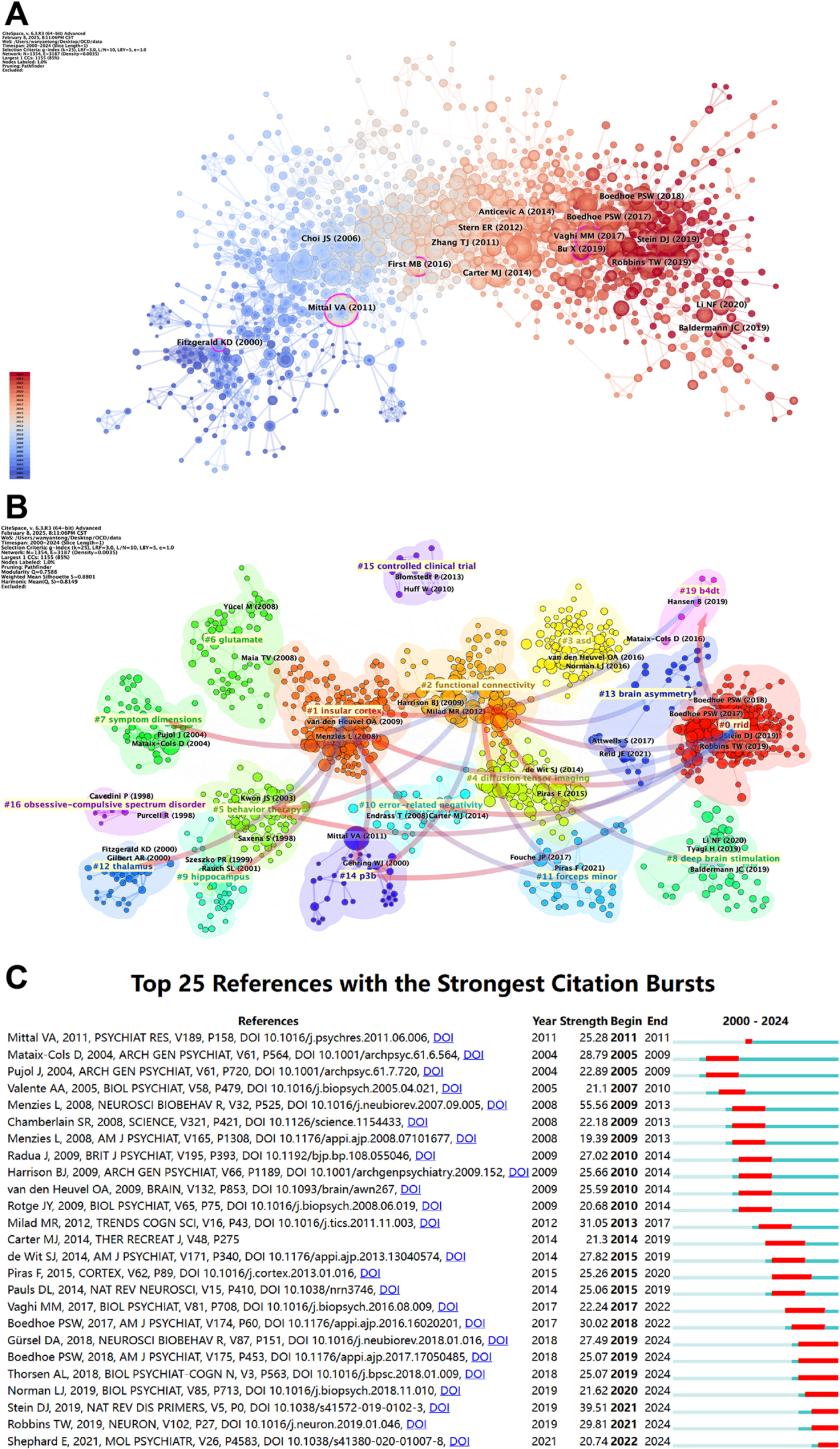
Highly cited references citation and co-citation analysis of OCD from 2000 to 2024. (**A**) A literature relation network diagram is generated using CiteSpace, where nodes of various colors represent different years, transitioning from blue for the earliest to red for the most recent. The size of each node is proportional to the frequency of references. Fuchsia-outlined nodes denote key articles with high betweenness centrality. (**B**) This figure organizes references by their similarity, with arrows indicating the direction of inter-cluster reinforcement. The magnitude of node size reflects co-citation frequency, and the links denote co-citation relationships. (**C**) The diagram identifies 25 key references, highlighted by red spikes along the timeline, each indicating a significant increase in citations. These peaks suggest pivotal moments in the field, possibly linked to the introduction of influential research or major breakthroughs

To more accurately evaluate the academic impact of articles, we utilized CiteSpace to analyze the temporal differences among highly cited articles and visualized the results in Fig. 9A. The nodes marked with purple circles in the figure represent key articles in the field of OCD that merit particular attention. These articles, arranged chronologically, include “Proton spectroscopic imaging of the thalamus in treatment-naive pediatric obsessive-compulsive disorder” [31], published in 2000 by Fitzgerald KD et al.; “Diagnostic and statistical manual of mental disorders” [32], published in 2011 by Mittal VA et al.; “The importance of developmental field trials in the revision of psychiatric classifications” [33], published in 2016 by First MB et al.; “Specific Frontostriatal Circuits for Impaired Cognitive Flexibility and Goal-Directed Planning in Obsessive-Compulsive Disorder: Evidence From Resting-State Functional Connectivity” [34], published in 2017 by Vaghi MM et al.; and “Investigating the predictive value of different resting-state functional MRI parameters in obsessive-compulsive disorder” [35], published in 2019 by Bu X et al. These articles have played a significant role in advancing research in the field of OCD.

Figure 9B illustrates the evolution of the knowledge structure within the OCD research field. High-impact publications were grouped into 20 thematic clusters based on co-citation patterns. Clusters that were weakly connected to the core network and did not contribute to the primary knowledge trajectory were excluded, while key research themes such as Research Resource Identifiers (RRID), brain asymmetry, and ASD were retained. Directional arrows indicate the progression of research topics over time, from earlier foundational themes to emerging research directions. For instance, Cluster #0 (RRID) contributed to the development of Cluster #2 (functional connectivity), which subsequently influenced Cluster #7 (symptom dimensions), reflecting a shift from methodological advances to network-based and clinically relevant frameworks.

Three clusters (#12 thalamus, #15 controlled clinical trial, and #16 obsessive–compulsive spectrum disorder) appear disconnected from the main co-citation network. Rather than indicating marginal relevance, this pattern reflects differences in research stage and functional role within the overall research pipeline. Specifically, the thalamus cluster represents early region-based studies that predate network-oriented frameworks; the obsessive–compulsive spectrum disorder cluster captures conceptual and diagnostic discussions that provide theoretical context rather than mechanistic insight; and the controlled clinical trial cluster corresponds to translational endpoints focused on evaluating intervention efficacy. The apparent isolation of these clusters suggests that their core concepts have been progressively incorporated into subsequent theoretical and methodological developments and therefore no longer emerge as independent research foci in co-citation-based analyses.

Figure 9C presents the citation burst analysis of the 25 most highly cited articles in the OCD field. The majority of these articles experienced peak citation periods lasting 3 to 5 years. Four articles had burst strengths exceeding 30. The article with the highest citation burst strength was a seminal review published in 2008 by Menzies L. in *Neuroscience & Biobehavioral Reviews*, entitled “*Integrating evidence from neuroimaging and neuropsychological studies of obsessive–compulsive disorder: the orbitofronto-striatal model revisited*.” This review represents a highly influential hub publication in the field of OCD neuroimaging, with a citation burst strength of 55.56 [36]. Ranking second was the article published in *Nature Reviews Disease Primers* in 2019 by DJ Stein et al. [1], with a burst strength of 39.51. The popularity of this article has continued through 2024 and is likely to attract further attention from researchers in the future. The third highest burst strength of 31.05 was achieved by “Obsessive-compulsive disorder: beyond segregated cortico-striatal pathways” [6], published in *Trends in Cognitive Science* in 2012 by MR Milad et al. Following closely was “Distinct Subcortical Volume Alterations in Pediatric and Adult OCD: A Worldwide Meta- and Mega-Analysis” [37], published in *American Journal of Psychiatry* in 2017 by PSW Boedhoe et al., with a burst strength of 30.02.

Notably, the work by Robbins et al. [3] and Gürsel et al. [38] exhibit strong and sustained citation bursts extending to 2024, suggesting that they represent two influential and complementary frontiers in contemporary OCD research. Importantly, these studies should not be interpreted as one replacing the other. Instead, they reflect parallel developments within the field: Gürsel et al. consolidate neuroimaging evidence within established network-based models, whereas Robbins et al. extend OCD research toward transdiagnostic and cognitive–neuroscientific frameworks. Together, they illustrate an ongoing shift from disorder-specific models toward broader theoretical integration.

## Discussion

### General information

This bibliometric analysis provides a comprehensive overview of the global research landscape in OCD neuroimaging from 2000 to 2024, revealing significant trends, knowledge gaps, and future directions. Our study highlights the progression of research themes, from early metabolic studies to contemporary network neuroscience, demonstrating the transformative impact of technological advancements such as fMRI, DTI, and AI-driven methodologies. Geographic disparities in collaboration networks were evident, with LMICs underrepresented despite comparable OCD prevalence. Keyword and citation analyses identified emerging frontiers like transdiagnostic biomarkers and neuromodulation therapies, while journal influence analysis revealed complementary roles for specialized and multidisciplinary platforms. These findings collectively offer insights into the current state of OCD neuroimaging research and underscore the need for greater equitable global collaboration to develop diagnostic and therapeutic strategies with broader applicability.

### Technological evolution and paradigm shifts in OCD neuroimaging

The trajectory of OCD neuroimaging research over the past 25 years reflects a dynamic interplay between technological innovation and theoretical refinement. Early investigations in the 2000 s, such as PET studies examining glucose metabolism [16], established foundational insights into metabolic dysregulation within the cortico-striatal pathways. The advent of fMRI and DTI, however, shifted focus toward functional and structural connectivity, exemplified by Menzies et al.’s (2008) integration of neuroimaging and neuropsychological data to refine the orbitofronto-striatal model [5]. Our keyword co-occurrence analysis (Fig. 7A) indicates the prominence of terms like “fMRI” (230 occurrences) and “functional connectivity” (72 occurrences), reflecting their dominance in contemporary research. This methodological shift has not only expanded investigative scope but also refined theoretical frameworks, enabling more nuanced exploration of OCD’s neurobiology.

The recent integration of AI and machine learning has further propelled the field toward data-driven, network-based approaches [39]. Since 2010, resting-state fMRI and AI-driven tools have surged in adoption, facilitating large-scale network analyses and transdiagnostic comparisons [40]. For example, Vaghi et al. demonstrated specific fronto-striatal circuit dysfunctions in OCD through resting-state functional connectivity [34], a finding corroborated by our citation burst analysis (Fig. 9C), which revealed sustained interest in “resting-state fMRI” since 2019. Machine learning applications, though nascent, show promise in addressing OCD heterogeneity through subtype-specific biomarker identification [14, 18]. The recent ENIGMA-OCD consortium study exemplifies this trend, employing machine learning to analyze white matter diffusion patterns across 1,653 individuals [19]. These advancements align with the growing influence of journals like *NeuroImage* and *Human Brain Mapping*, which prioritize methodological innovation and serve a key platform for disseminating cutting-edge neuroimaging research. This transition toward data-intensive, network-focused approaches signifies a broader paradigm shift, highlighting the critical role of interdisciplinary collaboration in unraveling OCD’s neurobiological complexity.

### Geographic and institutional collaboration

*Persistent Disparities and Emerging Networks.* Our analysis reveals significant geographical disparities in research output, with high-income countries (HICs) contributing most of publications. The United States accounted for 729 articles (36.5% of total output), whereas LMICs like Brazil and South Africa contributed minimally, despite comparable OCD prevalence rates [2]. This imbalance stems partly from resource limitations, as institutions in LMICs often lack advanced neuroimaging infrastructure. For example, the University of São Paulo published 25 articles, compared to Harvard University’s 67, yet its Faculdade de Medicina da USP has emerged as a collaborative hub through partnerships with U.S. and European institutions. Such collaborations demonstrate potential for mitigating resource gaps and fostering inclusivity. Our bibliometric analysis starkly reveals a profound geographical inequity in OCD neuroimaging research, characterized by the overwhelming dominance of HICs and a severe underrepresentation of LMICs. This representational bias poses a direct and substantial threat to the global generalizability of current neurobiological models of OCD, as findings derived almost exclusively from HIC populations may not account for the diverse genetic, environmental, and sociocultural contexts that influence the disorder’s expression worldwide. Brazil stands as a critical and instructive exception, demonstrating that achieving high-impact, globally integrated neuroimaging science is feasible in LMIC settings through strategic investment, capacity building, and sustained international collaboration [41]. To genuinely address this equity gap and enhance the validity of future discoveries, the field must prioritize proactive strategies. These include the intentional design of global consortia that incorporate LMIC sites from their inception, as well as the adoption of cost-effective and portable neurotechnologies to facilitate scalable data collection in diverse and underserved populations [42].

Western institutions dominate collaboration networks (Fig. 3A), raising concerns about the generalizability of findings. For instance, the ENIGMA-OCD Working Group [10, 11, 14], though global in scope, primarily recruit participants from HICs, potentially overlooking population-specific neurobiological variations. In contrast, China has seen a rapid increase in publication volume, ranking second with 265 articles (Fig. 5B), reflecting strategic investments in neuroscience. However, its late entry into international collaborations post-2015 suggests ongoing challenges in knowledge exchange. To address these inequities, initiatives such as the Global Mental Health Databank could promote data sharing and capacity-building in LMICs, fostering more inclusive research practices and enhancing the global relevance of OCD neuroimaging studies.

### Emerging frontiers: from circuit-based models to transdiagnostic biomarkers

The transition from metabolic studies to network neuroscience reflects a paradigm change in OCD research. Studies focusing on “glucose metabolic rates” exhibited a citation burst from 2000 to 2013 (Fig. 8B), yet subsequent work has prioritized systems-level analyses. This transition was catalyzed by the emphasis on distributed cortical networks beyond the traditional cortico-striato-thalamo-cortical (CSTC) model. For example, Milad and Rauch’s influential review has accrued 590 times [6], establishing the role of these networks in OCD. Our timeline (Fig. 7C) identifies emerging themes including “transdiagnostic biomarkers” (cluster #7) and “neuromodulation” (cluster #0), consistent with findings by Goodkind et al., who identified shared gray matter reductions across different psychiatric disorders [29].

DBS and AI-enhanced predictive modeling represent transformative therapeutic directions. The keyword “deep brain stimulation” (86 occurrences) predominantly links to clinical trials targeting the anterior limb of the internal capsule. Studies have shown promising results in reducing OCD symptoms, particularly in treatment-resistant cases [43, 44]. However, the underrepresentation of pediatric populations in these studies highlights a critical gap, given OCD’s frequent onset in childhood. A recent scoping review [45] of DBS in pediatric populations identified only 13 clinical trials, with most focusing on dystonia and epilepsy, indicating limited research on OCD in this age group. Importantly, the field is undergoing a paradigm shift from disorder-specific models toward a transdiagnostic framework focused on identifying shared neural substrates across psychiatric conditions. This shift is motivated by the recognition that core clinical dimensions, such as cognitive inflexibility, excessive threat monitoring, or altered reward processing, are present across traditional diagnostic categories including obsessive-compulsive disorder, anxiety disorders, and depression [29]. Consequently, research is moving beyond the CSTC circuit to map these dimensions onto broader brain networks. For instance, impairments in cognitive control are linked to dysfunction in frontoparietal networks also implicated in attention-deficit/hyperactivity disorder and major depressive disorder [46]. Similarly, abnormalities in the salience network are associated with atypical threat processing, a feature spanning multiple anxiety-related conditions [47]. Large-scale meta-analyses have begun to identify common brain structural alterations across multiple psychiatric diagnoses [29]. This transdiagnostic approach is crucial for discovering biomarkers that reflect fundamental neurobehavioral processes, promising to inform neurobiologically grounded classifications and therapies that target shared mechanisms.

Additionally, advances in AI and machine learning have facilitated predictive modeling of neuroimaging data. For instance, Bu et al. demonstrated the predictive utility of resting-state fMRI parameters in OCD prognosis, showing that specific neural signatures could inform clinical outcomes [48]. This approach has the potential to enhance personalized treatment strategies by identifying biomarkers associated with treatment response. However, further research is needed to validate these findings and expand their application to diverse populations, given OCD’s frequent onset in childhood [49].

### Methodological challenges and the imperative for reproducibility

Despite significant technological progress, methodological heterogeneity remains a barrier to achieving consensus in OCD neuroimaging research. Variability in fMRI preprocessing pipelines, for instance, complicates cross-study comparisons, as highlighted by Parkes et al. in their evaluation of motion correction strategies [50]. Our co-citation analysis indicates a heavy reliance on journals including *Biological Psychiatry* (5,435 citations) and *NeuroImage* (4,827 citations), although their analytic transparency guidelines show inconsistent adoption. Furthermore, the limited number of longitudinal studies limits insights into OCD’s neurodevelopmental progression, compromising our understanding of how neurobiological markers evolve over time.

The replication crisis in psychiatry demands urgent action. Only 5% of studies included raw imaging data, echoing Mitelman’s critique of the reproducibility challenges in neuroimaging research [17]. Although journals now require data availability statements, compliance remains inconsistent. Promising solutions include federated learning platforms, which facilitate multi-site analyses without data transfer—a strategy successfully implemented by the ENIGMA-OCD consortium [10, 11]. These approaches provide a pathway toward enhancing reproducibility and transparency, ensuring that advances in OCD neuroimaging are both robust and generalizable across diverse populations and research settings.

### Journal influence and interdisciplinary crossover

High-impact journals serve a dual function by disseminating cutting-edge findings and shaping research priorities. While journals such as *Biological Psychiatry* (IF 9.6, Q1) and *American Journal of Psychiatry* (IF 15.1, Q1) dominate citation networks, their broad scope often marginalizes OCD-specific research. Conversely, specialized journals like Journal of *Obsessive-Compulsive and Related Disorders* (IF 1.9, Q3) publish OCD-focused articles but struggle with visibility. This discrepancy highlights the need for balanced publishing strategies, a challenge addressed by journals like *NEUROCOMPUTING* (IF 5.5), which bridge AI and clinical neuroscience.

Interdisciplinary integration has become vital to progress in OCD research. The dual-map overlay (Fig. 6D) illustrates frequent citations spanning molecular biology to psychology journals, reflecting integrative approaches. For example, Stein et al.‘s (2019) primer in Nature Reviews Disease Primers [1] synthesized genetic, neuroimaging, and clinical data, epitomizing translational scholarship. However, computational psychiatry remains underrepresented in high-impact journals, indicating untapped potential for AI-driven advances. Collectively, these observations emphasize the importance of strengthening interdisciplinary collaboration to maximize the global impact of OCD research.

### Limitations and future directions

This study has several limitations. First, by focusing on English-language articles and excluding preprints, our dataset may have overlooked valuable contributions from researchers in LMICs. Second, bibliometric tools like CiteSpace tend to emphasize citation counts rather than methodological rigor, which could overestimate the impact of studies with methodological flaws. Third, the 2024 cutoff date might exclude emerging trends, such as the application of quantum computing in neuroimaging.

To address these limitations and advance the field, future research should expand funding initiatives for consortia led by LMICs, drawing inspiration from models like the NIH’s Fogarty International Center. Establishing standardized OCD-specific protocols for neuroimaging acquisition and analysis, endorsed by journals and professional organizations, would enhance methodological consistency. Longitudinal studies tracking neurodevelopmental changes in pediatric OCD groups are essential for understanding the early phases of the disorder. Promoting open science practices, such as mandatory data sharing on platforms like OpenNeuro, along with incentives to encourage LMIC participation, would ensure more comprehensive representation. Finally, integrating AI, particularly generative models for simulating OCD circuits and forecasting treatment outcomes, could significantly enhance research capabilities and their clinical application.

## Conclusions

This bibliometric analysis traces the evolution of OCD neuroimaging from a specialized field to a key component of translational research. Despite persistent geographical and methodological biases that threaten progress, emerging tools such as the COBIDAS (OHBM Committee on Best Practice in Data Analysis and Sharing) and federated learning, along with interdisciplinary collaboration, offer promising pathways toward equitable and reproducible research. By prioritizing global collaboration and standardization, the field can develop biomarkers and therapies that are effective and relevant across diverse populations, ensuring broader impact and clinical applicability.

## Supplementary Information

Supplementary Material 1

## Data Availability

The dataset compiled for this study is available upon reasonable request to the corresponding authors.
