# Supplementary material for: Global scientific trends on neuroimaging in obsessive-compulsive disorder in the early twenty-first century: a bibliometric analysis and visualization analysis
Source: Ann Gen Psychiatry. 2026 Mar 31;25:38. doi: 10.1186/s12991-026-00653-6 (PMC13159320; doi:10.1186/s12991-026-00653-6)
Supplement: Supplementary file 1 — Supplementary Material 1 [file 12991_2026_653_MOESM1_ESM.docx]

Table S1: Annual distribution of publications and citations on neuroimaging research in obsessive-compulsive disorder (2000–2024)

| **Year** | **Articles** | **Total Articles** | **Citations** |
| --- | --- | --- | --- |
| 2000 | 24 | 24 | 14 |
| 2001 | 17 | 41 | 64 |
| 2002 | 16 | 57 | 77 |
| 2003 | 37 | 94 | 206 |
| 2004 | 25 | 119 | 366 |
| 2005 | 48 | 167 | 617 |
| 2006 | 44 | 211 | 898 |
| 2007 | 48 | 259 | 1183 |
| 2008 | 57 | 316 | 1687 |
| 2009 | 64 | 380 | 1971 |
| 2010 | 49 | 429 | 2406 |
| 2011 | 76 | 505 | 2598 |
| 2012 | 89 | 594 | 3093 |
| 2013 | 95 | 689 | 3918 |
| 2014 | 108 | 797 | 4241 |
| 2015 | 89 | 886 | 4382 |
| 2016 | 103 | 989 | 4605 |
| 2017 | 87 | 1076 | 4787 |
| 2018 | 103 | 1179 | 4605 |
| 2019 | 149 | 1328 | 6043 |
| 2020 | 137 | 1465 | 6507 |
| 2021 | 155 | 1620 | 8457 |
| 2022 | 138 | 1758 | 7539 |
| 2023 | 123 | 1881 | 6559 |
| 2024 | 119 | 2000 | 7340 |
